# Supplementary material for: 24 h Activity Guidelines in Children and Adolescents: A Prevalence Survey in Asia-Pacific Cities
Source: Int J Environ Res Public Health. 2023 Jul 19;20(14):6403. doi: 10.3390/ijerph20146403 (PMC10379132; doi:10.3390/ijerph20146403)
Supplement: Supplementary file 1 [file ijerph-20-06403-s001.zip › ijerph-2465677-supplementary.pdf]

Table S1. Participating country WHO region World Bank classification.

Country	WHO region	World Bank classification	Local institution	Location of local institution in the city	Settings for data collection	Chief investigator
India	SEARO	Lower-middle income	Noorul Islam Institute Of Medical Science And Research Foundation (NIMS)	Thiruvananthapuram	Urban: Multiple cities in India	Muttathu KC Nair
Sri Lanka	SEARO	Upper-middle income	University of Kelaniya	Kelaniya	Urban: Kelaniya, Gampaha, Negombo	Sachith Mettananda
Hong Kong	WPRO	High-income	Queen Elizabeth Hospital	Kowloon	Urban: Hong Kong Island, Kowloon and the New Territories	Betty Wai-Man But
Singapore	WPRO	High-income	1)Centre for Fathering<br>2) Sembawang Primary School<br>3)Outram Secondary School<br>4)National University Hospital System<br>5)KK Women's and Children's Hospital	Singapore	Urban: Kent Ridge, Little India, Sembawang Outram	1)Michael Yong Hwa Chia<br>2)Teresa Shu Zhen Tan<br>3) Phaik Ling Quah
Japan	WPRO	High-income	Nihon University School of Medicine	Tokyo	Urban: Tokyo	Nobuhiko Nagano

South-east Asian region; WPRO, Western Pacific region.

Table S2. Baseline characteristics of participants by city.

Baseline characteristics	India (n = 126) n (%)	Kelaniya, Sri Lanka (n = 470) n (%)	Kowloon, Hong Kong (n = 192) n (%)	Singapore (n = 241) n (%)	Tokyo, Japan (n = 110) n (%)	p value
**Parental characteristics**						
Education						0.001
Primary or secondary	22 (17.5)	187 (39.8)	6 (3)	12 (5)	21 (19.1)	
Post-secondary	23 (18.3)	169 (36.0)	13 (7)	70 (29)	53 (48.2)	
University	81 (64.2)	114 (24.2)	173 (90)	159 (66)	36 (32.7)	
Participants						0.001
Mother	86 (68.25)	348 (74.04)	151 (78.65)	168 (69.7)	102 (92.73)	
Father	40 (31.75)	122 (25.96)	41 (21.35)	68 (28.2)	8 (7.27)	
Legal Guardian		0 (0)	0 (0.0)	5 (2.1)	0 (0)	
Age (year), mean (SD)	37.4(6.0)	37.1 (14.0)	43.3 (6.4)	42.5 (7.5)	40.1(7.6)	0.001
**Child's characteristics**						
Birth order						0.001
1st child	92 (73.0)	242 (51.5)	149 (77.6)	147 (61.0)	73 (66.4)	
2nd child	30 (23.8)	181 (38.5)	35 (18.2)	67 (27.8)	29 (24.4)	
3 child or more	4 (3.2)	47 (10.0)	8 (4.2)	27 (11.2)	8 (7.3)	
Gender						0.001
Male	65 (51.6)	197 (41.9)	90 (46.9)	137 (56.8)	60 (54.6)	
Female	61 (48.4)	273 (58.1)	102 (53.1)	104 (43.2)	50 (45.4)	
Chronic illness						0.001
No	121 (96)	449 (95.5)	181 (94.3)	229 (95.0)	94 (85.5)	
Yes	5 (4)	21 9 (4.5)	11 (5.7)	12 (5.0)	16 (14.5)	
Child age (years), mean (SD)	11.0 (10.7)	12.20 (3.6)	10.6 (3.9)	11.15 (3.9)	9.29 (3.1)	0.001

Missing data: BMI z-score (India=18, Kelaniya=112, Kowloon= 17, Singapore=13, Tokyo=20).

Table S3. Perception and awareness of child health and movement behaviours by city.

Perceptions and awareness	India (*n* = 126) *n* (%)	Kelaniya, Sri Lanka (*n* = 470) *n* (%)	Kowloon, Hong Kong (*n* = 192) *n* (%)	Singapore (*n* = 241) *n* (%)	Tokyo, Japan (*n* = 110) *n* (%)	*p* value
Perception of child weight						0.001
Overweight	13 (10.3)	54 (11.5)	27 (14.1)	36 (14.9)	13 (11.8)	
Underweight	9 (7.1)	19 (4.0)	27 (14.1)	48 (19.9)	20 (18.2)	
Normal weight	104 (82.5)	397 (84.5)	138 (71.7)	157 (65.2)	77 (70.0)	
Do you feel that your child receives adequate amount of physical activity to benefit his/her growth, development and health?						0.001
Yes	98 (77.8)	304 (64.7)	91 (47.4)	137 (56.9)	63 (57.3)	
No	28 (22.2)	166 (35.3)	101 (52.6)	104 (43.2)	47 (42.3)	
Are you concerned about the amount of recreational screen time your child is currently exposed to?						0.001
Yes	96 (96.2)	432 (91.9)	139 (72.4)	150 (62.2)	74 (67.3)	
No	30 (23.8)	38 (8.1)	53 (27.6)	91 (37.8)	36 (32.7)	
Do you think your child is getting adequate sleep to support his/her growth, development and health?						0.001
Yes	116 (92.1)	441 (93.8)	116 (60.4)	155 (64.3)	74 (67.3)	
No	10 (7.9)	29(6.2)	76 (39.6)	86 (35.7)	36 (32.7)	
Do you think your child is receiving adequate calories to support his/her growth, development and health?						0.001
Yes	106 (84.1)	413 (87.9)	140 (72.9)	182 (75.5)	69 (62.7)	
Yes, too much	5 (4.0)	25 (5.3)	31 (16.2)	25 (10.4)	12 (10.9)	
No	15 (11.9)	32 (6.8)	21 (10.9)	34 (14.1)	29 (26.4)	
Are you aware of the current recommendations regarding the amount of physical activity your child should be receiving per day?						0.001
Yes	102 (80.9)	175 (37.2)	125 (65.1)	107 (44.4)	15 (13.6)	
No	24 (19.1)	295 (62.8)	67 (34.9)	134 (55.6)	95 (86.4)	
Are you aware of the current recommendations regarding the amount of sleep your child should be receiving per day?						0.001
Yes	115 (91.3)	289 (61.5)	134 (67.8)	185 (75.8)	51 (46.4)	
No	11 (8.7)	181 (38.5)	58 (30.2)	56 (23.2)	59 (53.6)	
Are you aware of the current recommendations regarding the amount of screen time your child should be limited to per day?						0.001
Yes	101 (80.2)	195 (41.5)	135 (70.3)	126 (52.3)	28 (25.4)	
No	25 (19.8)	275 (58.5)	57 (29.7)	115 (47.7)	82 (74.6)	
